# Enhanced production of terrein in marine-derived *Aspergillus terreus* by refactoring both global and pathway-specific transcription factors

**DOI:** 10.1186/s12934-022-01859-5

**Published:** 2022-07-06

**Authors:** Guangshan Yao, Xinfeng Bai, Bingxin Zhang, Lu Wang, Songbiao Chen, Zonghua Wang

**Affiliations:** 1 School of Geography and Oceanography, Minjiang University, Fuzhou, 350108 China; 2 Key Laboratory of Pathogenic Fungi and Mycotoxins of Fujian Province, Fujian Agriculture and Forestry University, Fuzhou, 350002 China; 3 Shandong Provincial Third Hospital, College of Medicine, Shandong University, Jinan, 250031 China; 4 Southeastern Ecological Fragile Area Monitoring and Restoration Engineering Technology Innovation Center of the Ministry of Natural Resources, Fuzhou, 350000 China; 5 State Key Laboratory of Ecological Pest Control for Fujian and Taiwan Crops, College of Plant Protection, Fujian Agriculture and Forestry University, Fuzhou, China

**Keywords:** Terrein, *Aspergillus terreus* cell factory, Transcription factor, StuA, TerR, secondary metabolism

## Abstract

**Background:**

Terrein, a major secondary metabolite from *Aspergillus terreus*, shows great potentials in biomedical and agricultural applications. However, the low fermentation yield of terrein in wild *A*. *terreus* strains limits its industrial applications.

**Results:**

Here, we constructed a cell factory based on the marine-derived *A*. *terreus* RA2905, allowing for overproducing terrein by using starch as the sole carbon source. Firstly, the pathway-specific transcription factor TerR was over-expressed under the control of a constitutive gpdA promoter of *A*. *nidulans*, resulting in 5 to 16 folds up-regulation in *terR* transcripts compared to WT. As expected, the titer of terrein was improved in the two tested *terR* OE mutants when compared to WT. Secondly, the global regulator gene *stuA*, which was demonstrated to suppress the terrein synthesis in our analysis, was deleted, leading to greatly enhanced production of terrein. In addition, LS-MS/MS analysis showed that deletion of StuA cause decreased synthesis of the major byproduct butyrolactones. To achieve an optimal strain, we further refactored the genetic circuit by combining deletion of *stuA* and overexpression of *terR*, a higher terrein yield was achieved with a lower background of byproducts in double mutants. In addition, it was also found that loss of StuA (both Δ*stuA* and Δ*stuA*::OE*terR*) resulted in aconidial morphologies, but a slightly faster growth rate than that of WT.

**Conclusion:**

Our results demonstrated that refactoring both global and pathway-specific transcription factors (StuA and TerR) provides a high-efficient strategy to enhance terrein production, which could be adopted for large-scale production of terrein or other secondary metabolites in marine-derived filamentous fungi.

**Supplementary Information:**

The online version contains supplementary material available at 10.1186/s12934-022-01859-5.

## Introduction

( +)-Terrein, the major secondary metabolite produced by *Aspergillus terreus*, was firstly discovered in 1935 and its chemical structure was elucidated in the 1950s. Until recently, terrein has been reported to possess strong anti-tumor activities by targeting multiple pathways, including inhibition of melanin biosynthesis [1–3], anti-proliferation [4, 5], suppressing angiogenin production and inducing apoptosis of carcinoma cells [6]. And, terrein displays strong cytotoxicity against ABCG2-expressing breast cancer cells [7], further expanding its potentials in the development of effective therapeutic drugs of cancer. Also, terrein has anti-inflammatory properties in multiple cell lines [8–10], phytotoxicity [11], and antimicrobial activities [12]. Interestingly, terrein is the first reported dual inhibitor of QS and c-di-GMP signaling in the pathogenic bacteria *Pseudomonas aeruginosa* [13], showing potential in the development of novel antibiotics against multidrug-resistant (MDR) gram-negative bacteria. Therefore, terrein has important potential applications in medicine and agriculture.

**Fig. 1:**
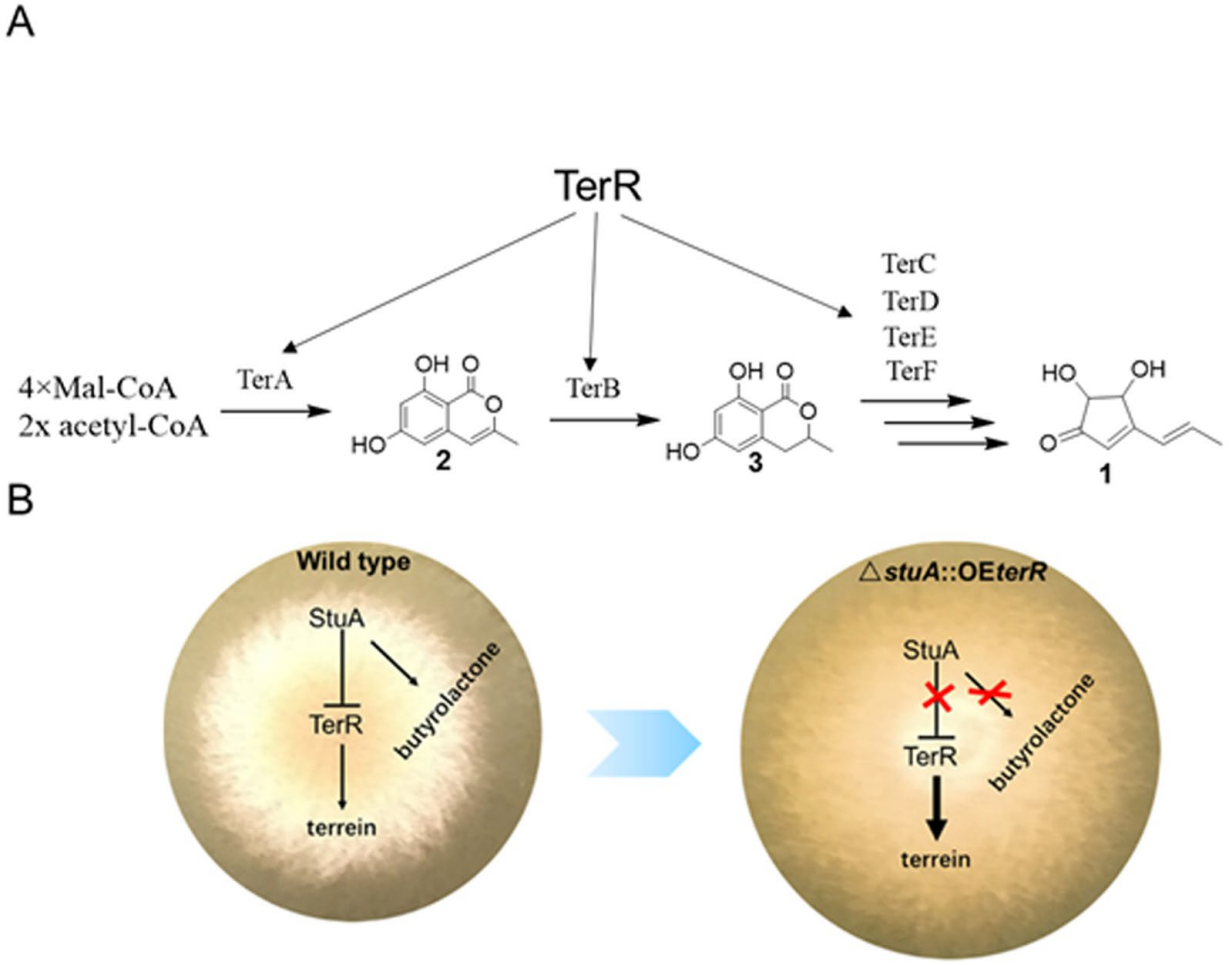
Graphical abstract and summary (**A**) biosynthesis of terrein (**B**) development of a cell factory for terrein production

The biosynthetic gene cluster for terrein has been identified, and its biosynthetic pathway has partially elucidated by genetic and biochemical analysis [11]. The cluster, which responsible for terrein synthesis, consists of 11 functional genes. Initially, two acetyl-CoA and four malonyl-CoA units are condensed and catalyzed by TerA, the non-reducing polyketide synthase, to generated 6-hydroxy-2,3-dehydromellein (2,3-dehydro-6-HM). Then, 2,3-dehydro-6-HM is reduced to 6-HM by the multi-domain protein TerB (Fig. 1A). Until now, the biosynthesis pathway from 6-HM to the end product of terrein remains unresolved. Markus Gressler et al. reported that nitrogen or iron starvation acts as the signal to induce terrein production, which depend on function of the global transcription factors AreA, AtfA and HapX [14]. Notably, elevated methionine level promotes terrein production even under non-inducing condition [14].

Like other fungal secondary metabolites, the production level is the bottleneck of further development and application of terrein. Screening of strains and media, and process optimization had been applied to improve terrein titers [15–18]. However, rational strain engineering has yet been used to improve terrein production until now. In this study, we achieved the high-producing and robust cell factory for terrein synthesis by modifying both the pathway-specific regulator TerR and the global regulator StuA (Fig. 1B). The modified mutants grow faster and produce terrein at gram level by using low-cost substance. We also found that the mutant strain can further reduce the costs by inhibition of biosynthesis of byproducts.

## Results

### Identification the biosynthetic gene cluster for terrein in marine-derived fungus *A*. *terreus* RA2905

( +)-Terrein is the major secondary metabolite produced by *A. terreus*, highlighted by its broad and excellent biological activities, including antimicrobial, anti-inflammatory, antitumor activities. Thus, its biosynthesis and regulatory mechanisms has always been a focus of attention in fungal secondary metabolism. A gene cluster of approximately 33 Kb, consisting of 11 functional genes, had been previously identified for terrein biosynthesis in *A*. *terreus* SBUG84411. By sequence alignment, we identified the terrein cluster in *A*. *terreus* RA2905 (Table 1). Overall, the terrein gene cluster was highly conserved between *A*. *terreus* RA2905 and *A*. *terreus* SBUG844. The exception is that a protein of 118 amino acids with unknown function specially exists in the terrein cluster of *A*. *terreus* RA2905, but not in *A*. *terreus* SBUG844. According to previous findings, the protein is not likely involved in biosynthesis of terrein. In the biosynthesis of terrein (Fig. 1A), TerA firstly assembles two acetyl-CoA and four malonyl-CoA to generate 6-hydroxy-2,3-dehydromellein (2). Then, the compound 2 is reduced to 6-hydroxymellein (3) by TerB. Other four unidentified enzymes (TerC, TerD, TerE and TerF) involve in the later biosynthesis pathway from 3 to the end product terrein (1). A Zn_2_Cys_6_-class transcription factor, named TerR, was presented in RA2905. It had been previously reported that TerR played a positive role in the regulation of expression of other terrein biosynthesis genes in SBUG844 [14].

**Table 1:** Terrein biosynthetic gene cluster

RA2905	NIH 2624	Identity (%)	Functional description
gene2371	TerA	96.52	Non-reducing polyketide synthase
gene2372	TerB	96.81	6-Hydroxymellein synthase
gene2373	TerC	99.26	FAD-dependent monooxygenase
gene2374	TerD	99.51	FAD-dependent monooxygenase
gene2375	TerE	98.58	Multicopper oxidase
gene2376	TerF	99.08	Kelch-like protein
gene2377	TerG	100.00	Efflux pump
gene2378			Unknown
gene2379	TerH	99.41	NAD-dependent epimerase/dehydratase
gene2380	TerI	100.00	Lactoylglutathione lyase-like protein
gene2381	TerJ	90.30	Efflux pump
gene2382	TerR	98.07	Transcription factor

To determine whether *A*. *terreus* RA2905 has the ability to synthesize terrein, compounds in the crude extracts from its rice cultures were chemically purified and structurally characterized. One of these compounds (30 mg) with an appearance of white power was obtained, and its molecular formula was determined as C_8_H_10_O_3_ by the HR–ESI–MS data at 309.2 [2 M + H]^+^, which was consistent with the formula of terrein reported previously. Moreover, our ^1^H and ^13^C NMR data further confirmed the obtained compound was terrein (Additional file 1: Figure S1 and Figure S2). Together, these results suggested that *A*. *terreus* RA2905 could synthesize terrein, but the production titer was low.

### Overexpression of pathway-specific transcription factor TerR to improve terrein yield

**Fig. 2:**
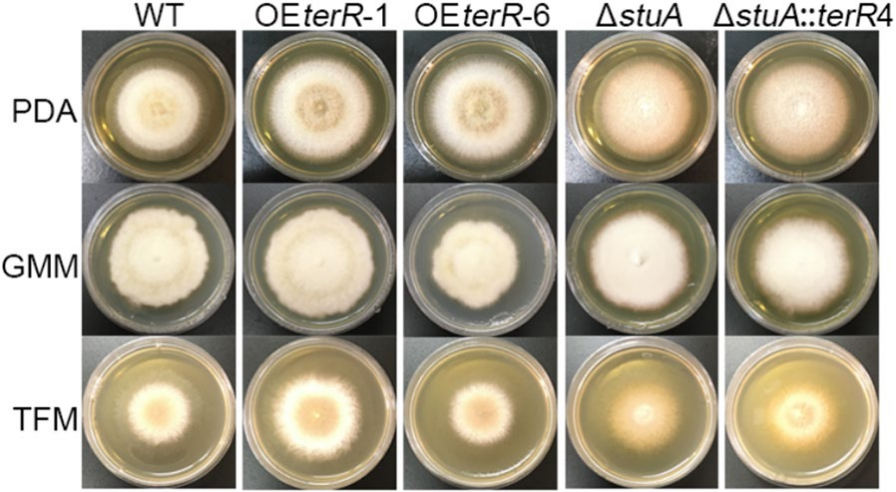
Phenotype and conidial development of terrein-producing mutant. All colonies were cultured on various media at 28 ℃ for 7 d

To activate the terrein cluster expression, firstly, we over-expressed the *terR* gene in RA2905. The constitutive and strong promoter from glyceraldehyde-3-phosphate dehydrogenase of *A. nidulans* was PCR-amplified and used to drive *terR* overexpression. Then, the *terR* expression cassette was fused with the selection marker gene *pyrg*, and then transformed into uracil autography mutant Δ*pyrG* established previously [19]. A total of 11 transformants with uracil prototroph were obtained, and five of them have the whole expression cassette by diagnostic PCR analysis. After further verification by using quantative PCR, OE*terR*-1 and OE*terR*-6 showing increased expression level in comparison to that of WT, were used for further analysis. The fungal phenotype and conidial development of two *terR* overexpression mutants and WT were analyzed (Fig. 2). The results showed that overexpression of *terR* has a faint effect on fungal growth and asexual development in *A*. *terreus* RA2905 (Fig. 2). The OE*terR*-6 mutant showed slightly faster growth rate than that of WT on solid plates (Fig. 2). Both *terR* overexpression mutants produced largely equal amount of conidia on all plates tested (Fig. 2).

**Fig. 3:**
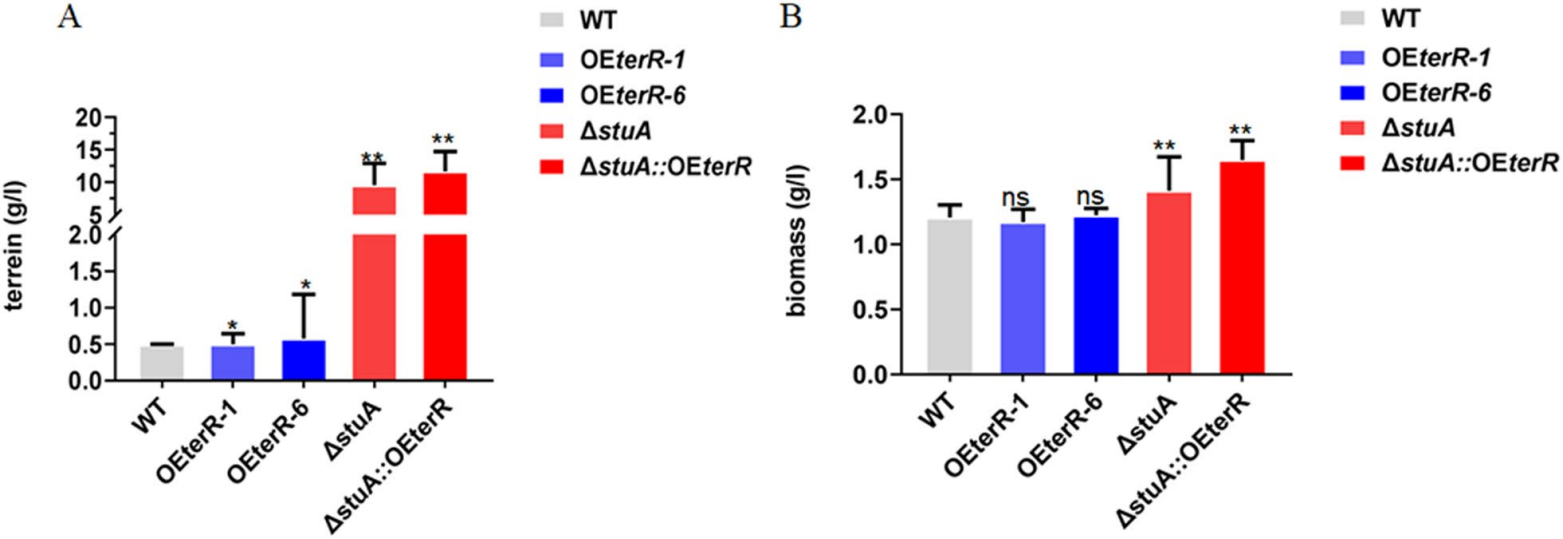
Yields of terrein and biomass in all mutant and WT strains. Fungal mycelia (10 g/L) were cultured in TFM media at 28 °C, 150 rpm for 7d, and yields of terrein and biomass for individual strain were determined by HPLC and dry weight method. Titer of terrein were the mean of three biological replicates. ns. means no significance, *p ≤ 0.05 and **p ≤ 0.01

By expected, the production of terrein was significantly improved in *terR* overexpression mutants compared to WT (Fig. 3A). Based on our preliminary data, a low-cost medium TFM, consisting of starch and peptone as carbon and nitrogen resource, was used for fermentation production of terrein. The 7-day fermentation, terrein were organically extracted and the titer was determined by HPLC. Our result showed that the titer of terrein was 0.5- and twofold improved in OE*terR*-1 and OE*terR*-6, respectively, as compared with that in WT strain (Fig. 3A). These results further demonstrated that TerR was a specific regulator of terrein, and its overexpression represented an efficient strategy to enhance terrein biosynthesis.

### Substantially enhancement of terrein yield by deleting *stuA*

In addition to pathway-specific regulator, transcription of most of secondary metabolite gene clusters are also controlled by global regulators. These regulatory proteins not only involve in secondary metabolism, but also have roles in fungal growth, development, as well as primary metabolic process [20]. The APSES transcription factor StuA was previously known as the conserved morphological modifier, playing crucial roles in cellular differentiation, mycelial growth, as well as asexual and sexual development in filamentous fungi [21, 22]. Recent studies has suggested that StuA regulates biosynthesis of multiple SM gene clusters in different species [21, 23], implying that it might function as a global regulator of SM in fungi. We presumed that StuA might contribute to biosynthesis of terrein in *A*. *terreus* for its high transcription abundance during terrein inducing media. The *stuA* mutant has been established in our previous investigation [19], but its biological function remain unknown in *A. terreus*.

As in other filamentous fungi, there was little to no conidia could be observed in the Δ*stuA* mutant regardless of media used (Fig. 2), while WT produced abundant amounts of conidia on any medium. Unlike in other fungi, our results suggested that radial growth of the Δ*stuA* mutant was approximately 10% increased as compared with that of WT on PDA, GMM, and TFM. Interestingly, a pale yellow pigment was clearly observed in the Δ*stuA* mutant, especially on the TFM plate, but not in WT. Considering that multiple precursors in the terrein biosynthesis are yellow, it can be speculated that the terrein gene cluster was activated in the mutant. To investigate the effect of StuA on terrein production, both the Δ*stuA* mutant and WT strains were cultured in TPM media to induce terrein biosynthesis. The yield of terrein was detected at 5 day by HPLC. The results showed that the Δ*stuA* mutant produced 10.8 mg/ml terrein, which was 21 folds more than that of WT. These results showed that deletion of *stuA* greatly enhanced terrein production.

### Construct a robust and high-producing cell factory for terrein synthesis by combing *terR* overexpression and *stuA* deletion

To further improve the terrein production, the pathway-specific transcription factor TerR and the global regulator StuA were simultaneously genetically modified in the marine-derived fungus *A*. *terreus* RA2905. The double gene mutant Δ*stuA*::OE*terR* were obtained by deletion of *stuA* in the OE*terR*-6 mutant with *hph* was used as the selectable marker gene described previously [19]. The resultant mutant Δ*stuA*::OE*terR* also lost the ability to develop conidia, which is the same as Δ*stuA* (Fig. 2), its terrein yield was further enhanced than that of Δ*stuA* or OE*terR*, reach the level of 12.6 g/L. More intriguingly, approximately 80% of fungal extract for Δ*stuA*::OE*terR* cultured in TFM media was terrein, which represent the highest yield to date.

### Loss of StuA improve the biomass of *A*. *terreus*

To investigate the TerR and StuA on fungal growth under fermentation process, the biomass of the Δ*stuA* and OE*terR* mutants, as well as WT strains were analyzed by dry cell weight assay (DCW) (Fig. 3B). Considering that the Δ*stuA* mutant did not produce conidia, a shift experiment was designed to achieve the same initial inoculum dose. The results showed that two *terR* overexpression mutants develop the same biomass as that of WT, suggesting that TerR not involved in fungal growth. In contrast, the biomass of Δ*stuA* mutant (16 g/L) was significantly increased compared to WT (12 g/L), indicating that StuA play a negative regulatory role in fungal growth.

### Expression analysis of terrein biosynthesis genes

**Fig. 4:**
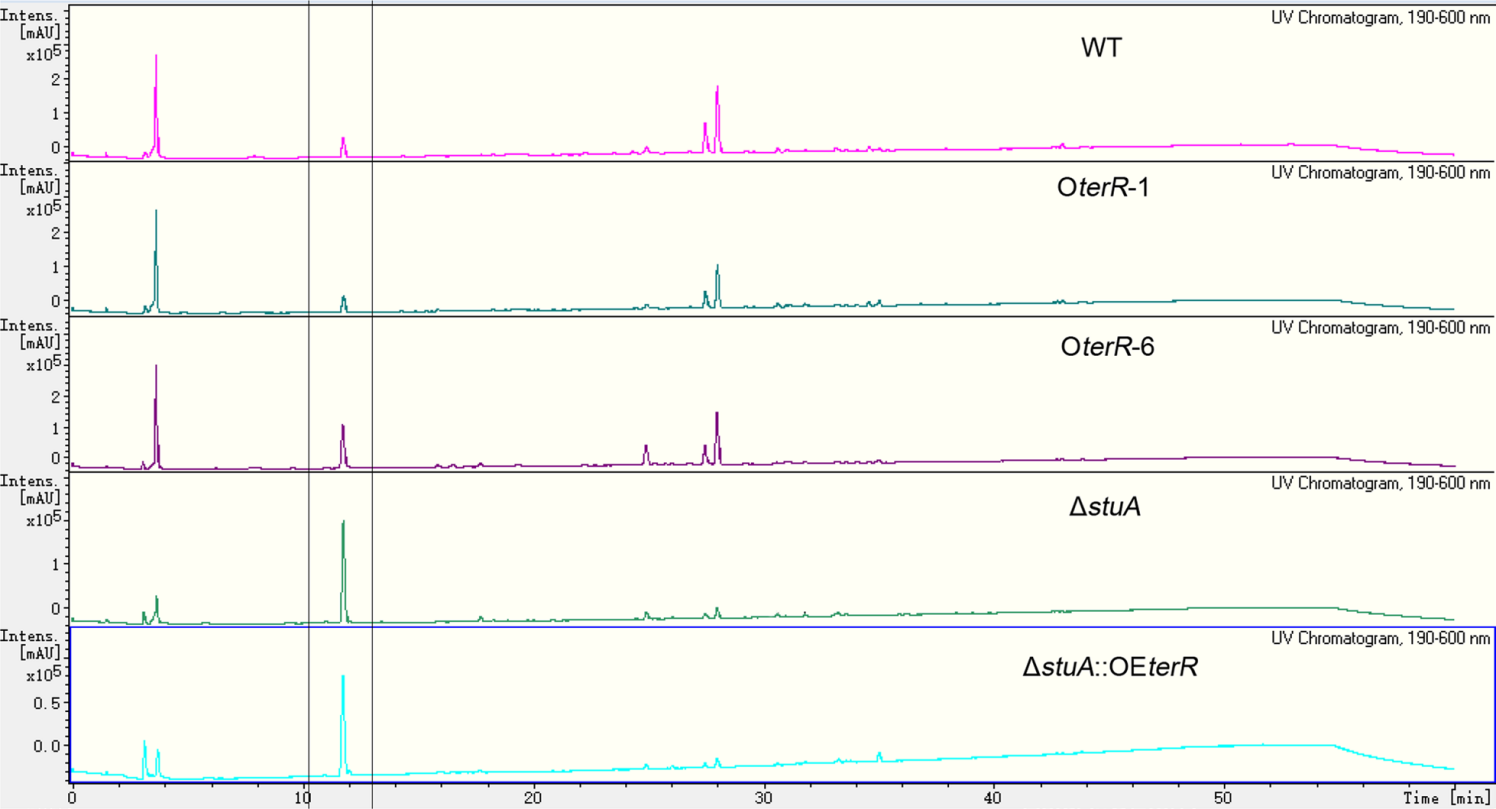
Expression level analysis of biosynthetic genes for terrein. Fungal strains were cultured in TFM media at 28 °C, 150 rpm for 48 h, and the gene expression of terA ~ terR were analyzed by semi-quantative PCR, and the expression level of actin gene was used as the control

Compared with the WT strain, a significant improvement in terrein yields was observed in OE*terR*, Δ*stuA*, Δ*stuA*::*terR* mutant mutants. We wondered whether this improvement was the result from up-regulation in gene transcription level. Therefore, the transcripts of the major terrein biosynthetic genes, including terA ~ *terR*, are analyzed by semi-quantitative PCR. Considering that no conidia were produced in Δ*stuA* or Δ*stuA*::*terR*, a shift experiment of mycelia was designed to ensure the same inoculation size and time among *stuA* mutants and WT. As expected, the results indicated that the transcript abundance of most of terrein biosynthetic genes were up-regulated significantly in both OE*terR* and Δ*stuA* mutant than that of WT under terrein induction media (Fig. 4), and the double mutant showed the highest expression level, which was consistent with terrein titer analysis above. These results demonstrated that all of these mutants improve the terrein production by upregulating the expression level of terrein biosynthetic genes.

### Block the biosynthesis of other secondary metabolites in terrein-producing mutant

**Fig. 5:**
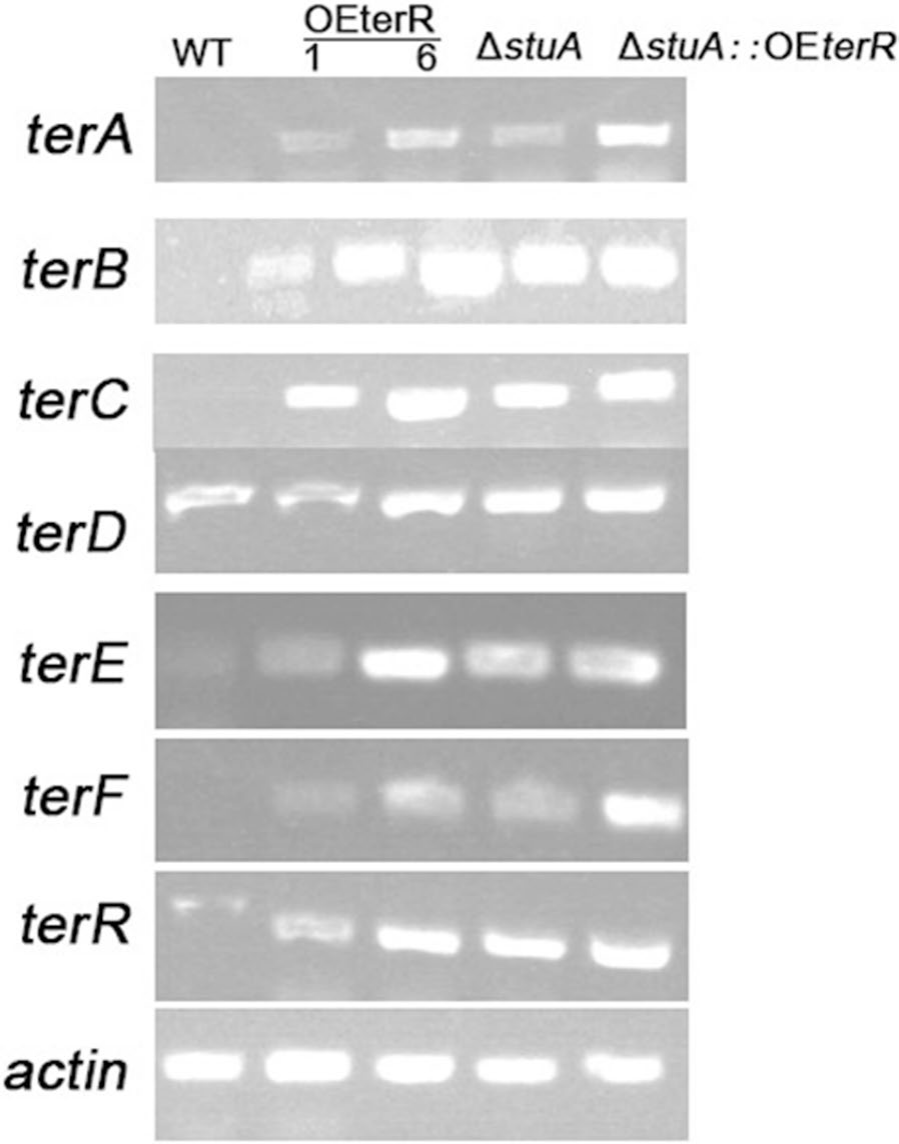
LC–MS/MS analysis of the extracts of both mutants and WT strains. Peak between two lines was confirmed to be the terrein based its molecular ion peak in MS

As a prolific producer in secondary metabolism, *A*. *terreus* wild type strain was reported to produce diverse secondary metabolites simultaneously. And, a variety of secondary metabolites, including butyrolactones, thiodiketopiperazine, and benzyl furanones and pyrones were isolated from the marine-derived *A*. *terreus* RA2905 [24, 25]. To determine the effect of *terR* overexpression, *stuA* deletion and combined mutation on the biosynthesis of other secondary metabolite, the extracts of OE*terR*, Δ*stuA* or Δ*stuA*::OE*terR* and WT were analyzed by LC–MS/MS (Fig. 5). The results further supported that the production of terrein in all mutants were enhanced, and Δ*stuA*::OE*terR* showed the highest yield, in turn by Δ*stuA*, and OE*terR* mutants. As expected, OE*terR* mutant produced comparable butyrolactones and other secondary metabolites as WT strain. Significantly, other secondary metabolites, specially butyrolactone, which was the major metabolite in WT, were completely abolished in Δ*stuA* or Δ*stuA*::OE*terR*. Therefore, Δ*stuA*::OE*terR* established here was a cell factory for robust and high-level producing terrein, which greatly reduced the following purification process.

## Discussion

The *A*. *terreus* secondary metabolite terrein had been demonstrated to possess extensive biological and pharmacological benefits, including anti-cancer, anti-inflammatory and antimicrobial activities. Furthermore, Matthias Brock et al. has reported that the terrein gene cluster was a high-performance expression system for biosynthesizing other fungal secondary metabolites [26]. However, the terrein yield and expression level of terrein genes were relatively low in the wild type strain of *A. terreus*. Therefore, identification and engineering of critical factor for terrein production is essential for providing resource of pharmacological research, as well as developing the more efficient and robust expression platform of fungal secondary metabolite gene cluster.

The APSES transcription factor StuA was initially found to be a regulator of fungal asexual and sexual development [22]. Recently, its regulatory role in secondary metabolism has been revealed in several fungi [21, 23]. In general, StuA positively regulates the SM gene expression, and its deletion resulted in reduction or even absence of secondary metabolite biosynthesis, such as aflatoxin [21]. However, it was interestingly found that deletion of *stuA* in *A*. *terreus* lead to reduced biosynthesis of other secondary metabolism, such as butyrolactones, but increase terrein production. However, the underlying mechanism remain further investigation.

Biosynthesis of secondary metabolites are controlled by both global and pathway-specific regulators in fungi. Global regulators, such as methyltransferase LaeA, were mainly more conserved than that of these pathway-specific regulators in the regulation of secondary metabolism [27, 28]. However, it was found that these global regulators also interfere with primary metabolic process in fungi, such as growth and conidial development, which caused unexpected defects [29]. While pathway-specific regulators are confined to the given BGC in a species, its function was specialized, could not deter the primary metabolism, as well as other secondary metabolite biosynthesis. Furthermore, these global regulators have regulatory roles in the expression of pathway-specific transcription factors, which establish a regulatory cascade for accurately and sharply control the secondary metabolite production. Thus, interpretation and engineering of the pathway-specific and global regulators, as well as its relationship, were essential for developing the efficient cell factory for a given and valued secondary metabolite production. Only alteration of either was limited for SM production engineering. Here, the regulatory program was redesigned by combing the pathway-specific regulator *terR* overexpression and the global regulator *stuA* deletion, the double mutant produced about 30 folds more terrein than that of WT, achieving ten-gram-level fermentation production of the anti-cancer compound terrein.

In conclusion, a genetic roadmap for engineering terrein production was established by combing redesign of both global and pathway-specific regulators, greatly improve the terrein production, not only help deepen the understand of regulatory mechanism of secondary metabolism, but provide a paradigm for yield improvement of other valued secondary metabolites in *A*. *terreus* and other filamentous fungi.

## Materials and methods

### Strains, primers and media

Strains of marine-derived *A*. *terreus* used in this study were listed in Table 2. All *A. terreus* strains were cultured on PDA (potato broth 20% and glucose 2% in sea water) media for propagation. GMM (Czapek–Dox salt 2% and glucose 2% in sea water) supplemented with 1 M sorbitol were used to for protoplast regeneration, or supplemented o.5 mM uridine and 1 mM uracil if necessary. TFM (starch 3%, peptone 1.5% and aginomoto 0.5%) media was used to ferment production of terrein. *A*. *terreus* mutants and WT strains were grown on PDA, GMM and TFM media supplemented with 1.5% agar for growth and conidial development analysis.

**Table 2:** Strains used in this study

Strain	Genotype	Reference
RA2905	WT	CCTCC AF 2,021,052
Δ*pyrG*	Δ*pyrG*::*ptrA*	[19]
OEterR-1	Δ*pyrG*::*ptrA*;P^*gpdA*^::*terR*::*AfpyrG*	This study
OEterR-6	Δ*pyrG*::*ptrA*;P^*gpdA*^::*terR*::*AfpyrG*	This study
Δ*stuA*	Δ*pyrG*::*ptrA*;Δ*stuA*::*AfpyrG*	[19]
Δ*stuA*::OE*terR*	Δ*pyrG*::*ptrA*;P^*gpdA*^::*terR*::*AfpyrG*	This study

### Construction of mutants

To construct *terR* overexpression strains, open reading frame (ORF) and terminator of *terR* was amplified from *A*. *terreus* genomic DNA with primers GPD*terR*F and *terR*1. The constitutive promoter of gene *gpdA* from *A. nidulans* was amplified from plasmid pIG1783 with primer pair GPDF/ GPDR, and the selectable maker gene *pyrG* as amplified from *A. fumigatus* genomic DNA with primers PyrGF/PyrGR. The three fragments obtained above were fused by using Double-Joint PCR based on the homologous sequence in primers. Preparation of protoplast and transformation of *A*. *terreus* RA2905 were performed as established previously [19]. All primers used were listed in Table 3.

**Table 3:** Primers used in this study

Name	Sequence	Use
PyrGF	GCCTCAAACAATGCTCTTCACCC	Amplification of *pyrG*
PyrGR	CAGAAAGAGTCACCGGTCACTGT ACGTCTGAGAGGAGGCACTGATGC	Amplification of *pyrG*
GPDF	GTACAGTGACCGGTGACTCTTTCTG	Amplification of promoter
GPDR	GGTGATGTCTGCTCAAGCGGGGTAG	Amplification of promoter
terR2	TCCCTTCATACCTCGCTTTA	Amplification of *terR*
GPDterRF	CCGCTTGAGCAGACATCACCATGTTCGCCGAACTTAACGC	Amplification of *terR*
terR1	TTTGAGGCTTATACAACGAG	Amplification of *terR*
terR2	AGACGCTTGTAGCCCGTTAT	Amplification of *terR*
TerARTF	AGCAGCAAGTCATACAGCAA	Expression analysis
TerARTR	AACTCCTCGCATAACAAAGC	Expression analysis
TerBRTF	CAAGGATTGAAGGCGGGATC	Expression analysis
TerBRTR	AGAAGTTTCGGGCACAGGTT	Expression analysis
TerCRTF	GCATCTGCAAAGCCTCCTGT	Expression analysis
TerCRTR	CTCTGGCGTGTCTCATACCG	Expression analysis
TerDRTF	TCAGGCTGGAGACAGTAATC	Expression analysis
TerDRTR	AACGCATCTGGTATCTGGTC	Expression analysis
TerERTF	TGGTGCCTCCCGAGAAACAG	Expression analysis
TerERTR	CTCGCCGACCTTGGCTACAT	Expression analysis
TerFRTF	TACGACCAGGAAGGAACAGT	Expression analysis
TerFRTR	GAGCCGATAGTAGCAGAAGC	Expression analysis
TerRRTF	CCCGACTCAGCAAGACGAAG	Expression analysis
TerRRTR	TGAAAGCCGAAAGCCATCAC	Expression analysis

### HPLC analysis of terrein

All strains were cultivated in TFM at 30 °C, 150 rpm for 7 days. The fungal cultures were extracted with ethyl acetate at least three times. HPLC analysis was performed on an Agilent InfinityLab LC Series 1260 system coupled with a Diode Array Detector WR detector and using an analytical Kromasil column (250 mm × 4.6 mm, 5 mm). The mobile phase was consisted of 0.1% formic acid in acetonitrile (A) and 0.1% formic acid in water (B), and the flow rate was 1 mL/min with 10 μL injection volume. Terrein was recorded at 281 nm.

### LC–MS analysis of secondary metabolites

LC–MS/MS were analyzed on a UHPLC system (1290, Agilent Technologies) with a UPLC HSS T3 column (1.8 μm 2.1 × 100 mm, Waters) coupled to a quadrupole time-of-flight mass spectrometer 6545 (Q-TOF, Agilent Technologies) equipped with an ESI dual source in positive-ion mode. The ESI conditions were as follows: the capillary temperature at 350 °C, source voltage at 4 kV, and a sheath gas flow rate of 0.5 mL/min. The mass spectrometer was operated with an auto MS/MS mode. Mass spectra were recorded from m/z 200 to m/z 1000 in a speed of 6 spectra/sec, followed by MS/MS spectra of the twelve most intense ions from m/z 200 to m/z 1000 in a speed of 12 spectra/sec.

### Gene expression analysis

All utilized *A. terreus* strains were inoculated into GMM liquid media and pre-cultured for 24 h, and then transferred into fresh LFM media at 28 °C for 48 h. The mycelia were collected via filtering, were then frozen in liquid nitrogen, and stored under − 80 °C conditions. RNA extraction and cDNA synthesis were performed using a RNA reagent (TRIzol reagent, Biomarker Technologies, Beijing, China) and a cDNA synthesis Kit (TransGen, Beijing, China) following the protocols of the manufactures, respectively. The quality and integrity of RNA samples was determined using Nanodrop 2000 (Thermo Fisher Scientific, USA) and Agilent 2100 bioanalyzer (Agilent Technologies, Palo Alto, CA), respectively, while the quantity was determined with a Qubit RNA assay kit (Life Invitrogen, USA). All utilized primers were listed in Table 3. The expression level of actin gene was used as the internal control.

## Supplementary Information

**Additional file 1:** Determine the structure of terrain. **Figure S1.** ^1^H NMR of terrain. **Figure S2.** ^13^C NMR of terrain.

## Data Availability

All data generated and analyzed during this study were included in this manuscript and the additional files.

## Abbreviations

gpdA: Glyceraldehyde 3-phosphate dehydrogenase
WT: Wild type
SM: Secondary metabolism
LS-MS/MS: Liquid chromatography tandem-mass spectrometry
QS: Quorum sensing
c-di-GMP: Cyclic-di-GMP
MDR: Multidrug-resistant
HR–ESI–MS: High-resolution electrospray ionization mass spectrometry
NMR: Nuclear magnetic resonance spectroscopy
HPLC: High performance liquid chromatography
