# Supplementary material for: Enhanced production of terrein in marine-derived Aspergillus terreus by refactoring both global and pathway-specific transcription factors
Source: Microb Cell Fact. 2022 Jul 6;21:136. doi: 10.1186/s12934-022-01859-5 (PMC9258105; doi:10.1186/s12934-022-01859-5)
Supplement: Supplementary file 1 — Additional file 1: Determine the structure of terrain. Figure S1. 1H NMR of terrain. Figure S2. 13C NMR of terrain. [file 12934_2022_1859_MOESM1_ESM.docx]

Detail structure information of terrein:

[α]20D +123.2 (c 0.5, MeOH); 1H NMR (500 MHz, DMSO-d6): 6.72 (1H, dq, J = 15.9, 6.9 Hz, H-7), 6.37 (1H, dd, J = 15.9, 1.9 Hz, H-6), 6.01 (1H, s, H-2), 5.80 (1H, d, J = 7.5 Hz, 5-OH), 5.69 (1H, d, J = 6.5 Hz, 4-OH), 4.50 (1H, dd, J = 7.3, 2.7 Hz, H-5), 3.88 (1H, dd, J = 6.4, 2.7 Hz, H-4), 1.88 (3H, dd, J = 6.9, 1.7 Hz, H-8). 13C NMR (125 MHz, DMSO-d6): δC 204.5 (C, C-1), 169.1 (C, C-3), 139.9 (CH, C-6), 125.1 (CH, C-2), 124.6 (CH, C-7), 81.1 (CH, C-5), 76.8 (CH, C-4), 19.1 (CH3, C-8). ESI-MS m/z 309.2 [2M + H]+。


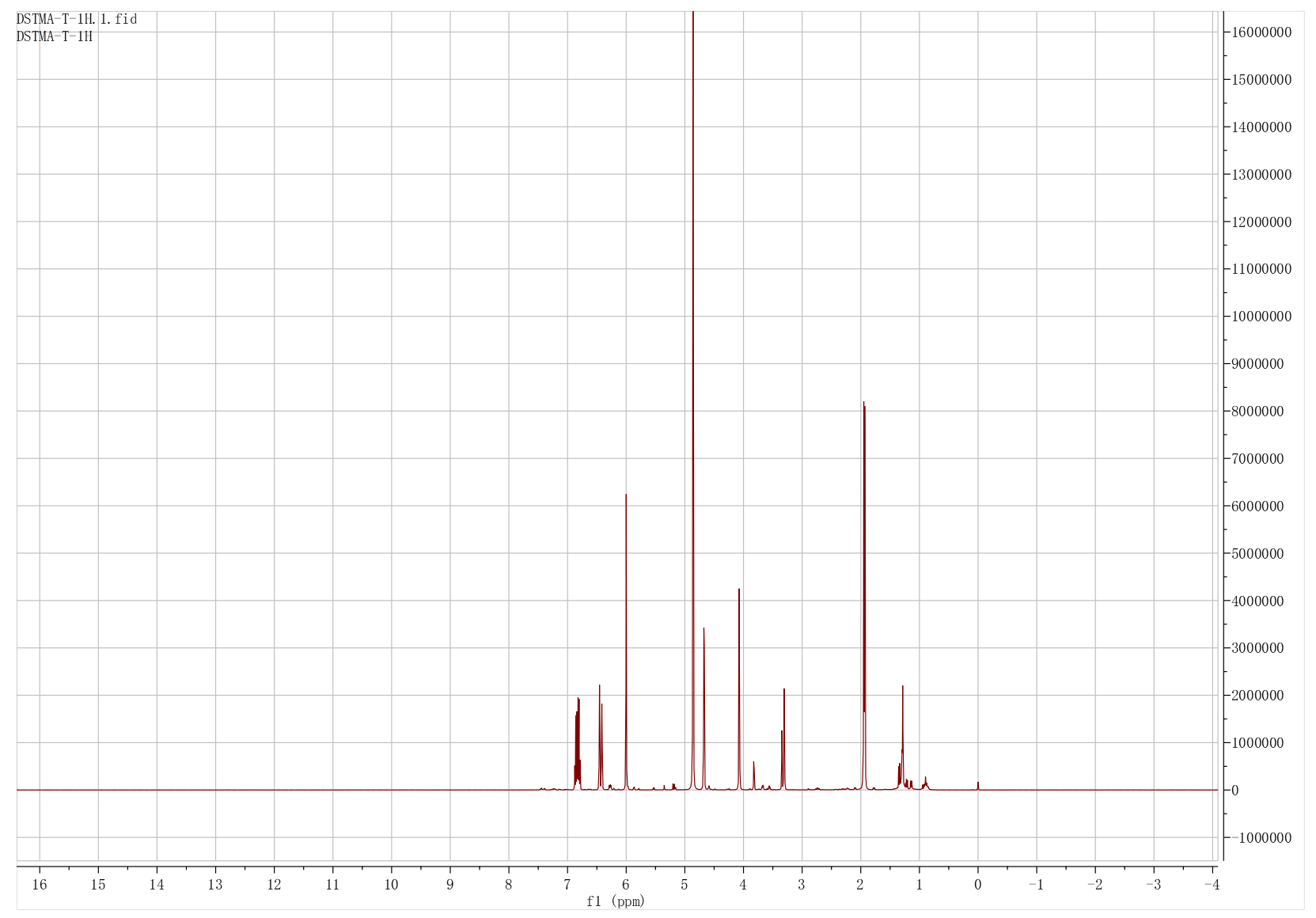


Fig. S1 ^1^H NMR of terrein


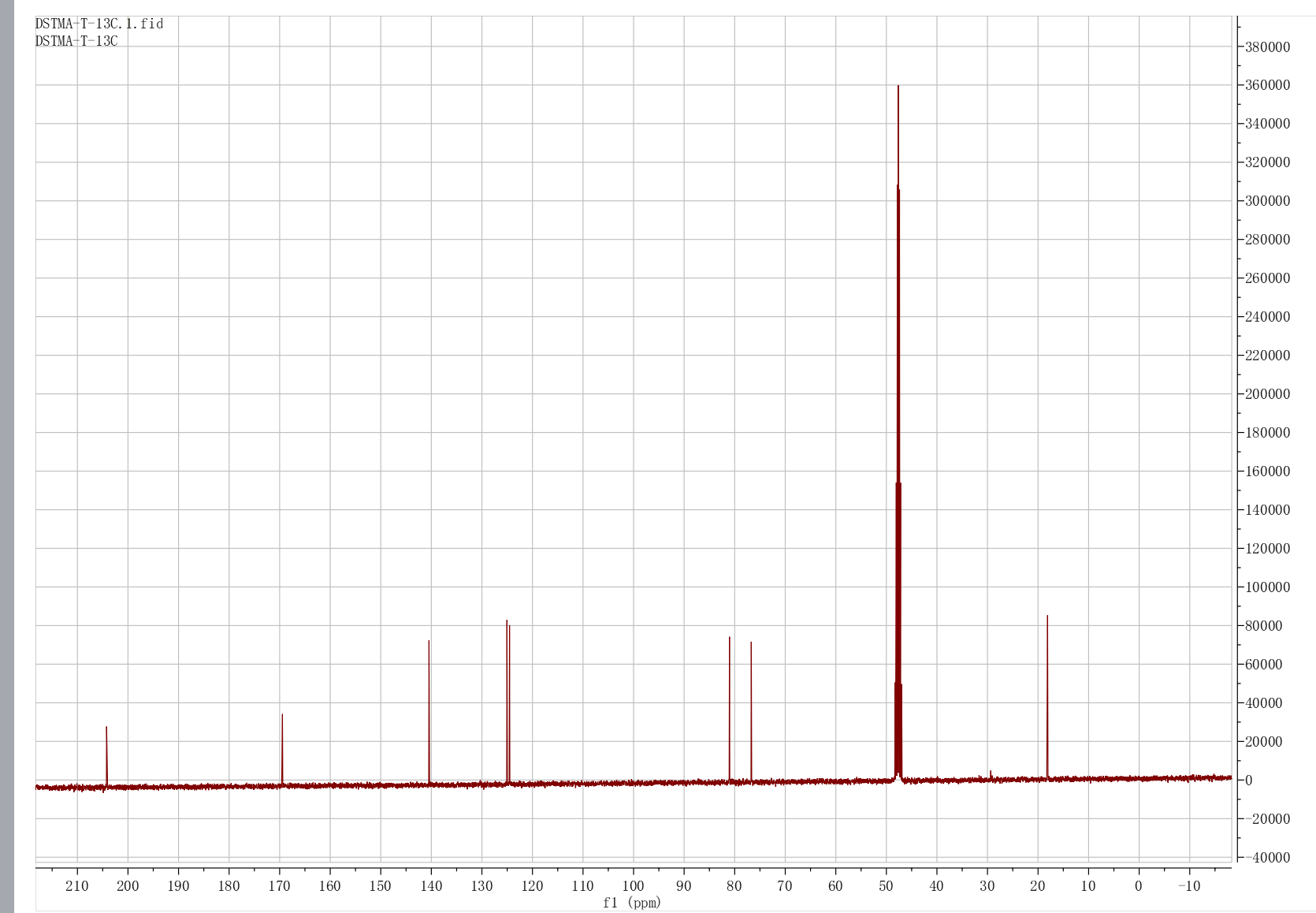


^13^C NMR of terrein
